# Maternal Low-Protein Diet Leads to Mitochondrial Dysfunction and Impaired Energy Metabolism in the Skeletal Muscle of Male Rats

**DOI:** 10.3390/ijms252312860

**Published:** 2024-11-29

**Authors:** Vipin A. Vidyadharan, Ancizar Betancourt, Craig Smith, Chellakkan S. Blesson, Chandra Yallampalli

**Affiliations:** 1 Basic Sciences Perinatology Research Laboratories, Department of Obstetrics and Gynecology, Baylor College of Medicine, Houston, TX 77030, USA; vidyadha@bcm.edu (V.A.V.);; 2 Agilent Technologies Inc., Santa Clara, CA 95051, USA; 3 Reproductive Endocrinology and Infertility Division, Baylor College of Medicine, Houston, TX 77030, USA; csblesson@bcm.edu; 4 Family Fertility Center, Texas Children’s Hospital, Houston, TX 77030, USA

**Keywords:** mitochondria, fetal programming, skeletal muscle, energy metabolism, low-protein diet, mitochondrial dynamics, mitochondrial dysfunction

## Abstract

A prenatal low-protein (LP) diet disrupts glucose homeostasis in adult offspring. Skeletal muscles are one of the main sites of glucose clearance, and mitochondria residing in the muscle fibers are central to glucose homeostasis. Our previous studies indicated that impaired mitochondrial health is central to dysregulated glucose metabolism in the gastrocnemius muscle of the LP-programmed female rats. In addition, dysfunctional mitochondria are often an indicator of underlying irregularities in energy metabolism and metabolic inflexibility. Therefore, this study examined the mitochondrial function and metabolic flexibility in the skeletal muscles of prenatal LP-programmed adult male rats. Pregnant Wistar rats were randomly allotted to a control diet (20% protein) or an isocaloric LP diet (6% protein). Standard laboratory rat chow was given to the dams and the pups after delivery and weaning. Gene and protein expressions, mtDNA copy number, and electron microscopy were assessed in gastrocnemius (GS) muscle, and the mitochondrial oxygen consumption rate was determined using isolated flexor digitorum brevis muscle fibers. The genes associated with mitochondrial outer membrane fusion, mitofusin1 and 2 (Mfn1 and Mfn2), fission (Fis1), and biogenesis (Pgc1B, Nrf1, and Esrra) were lower in the LP group. Further, our functional studies showed that the ATP-linked oxygen consumption rate (OCR), maximal, spare respiratory, and non-mitochondrial respiration-associated OCRs were lower in the LP rats. Further, the mRNA and protein expressions of Ndufb8, a key factor involved in the complex-I catalytic activity, were downregulated in the LP group. In addition, the expression of genes linked to mitochondrial pyruvate transport (Mpc1) and metabolism (Pdha1) was lower in the LP group. In contrast, the expression of mitochondrial fatty acid transporters (Cpt1a and Cpt2) was higher in the LP when compared to the control group. However, electron microscopic analysis exhibited no difference in the mitochondrial ultrastructure in the LP muscle compared to the control. Altogether, our results indicate that the LP diet affects the mitochondrial complex-I integrity and dynamics and leads to altered expression of genes associated with substrate oxidation and mitochondrial dysfunction in the skeletal muscle of the male LP offspring.

## 1. Introduction

Skeletal muscles are made up of bundles of muscle fibers, and they are one of the primary sites of insulin-stimulated glucose disposal in mammals. The health of mitochondria present in the skeletal muscle affects its overall glucose oxidation capacity [1,2,3]. Among different factors that contribute to mitochondrial health, balanced mitochondrial dynamics, normal mitochondrial morphology, effective energy metabolism, and metabolic flexibility are the key signs of healthy mitochondria [4,5]. Further, these factors are interlinked, and thus, minor changes in any of the factors could affect different mitochondrial functions. Thus, these factors are often impaired in dysfunctional mitochondria seen in the skeletal muscle and are associated with metabolic inflexibility and insulin resistance [6].

The dynamic nature of mitochondria is regulated by the molecules associated with mitochondrial fission, fusion, and biogenesis. The two key molecules involved in the fission process are dynamin-related protein-1(Drp1) and mitochondrial fission 1 protein (Fis1). The fusion process is a sum of outer and inner mitochondrial fusion with the outer membrane fusion regulated by mitofusin1 and 2 (Mfn1 and Mfn2), and the inner membrane fusion regulated by optic trophy 1 (Opa1), a dynamin-related GTPase [7]. The main markers of mitochondrial biogenesis are the mtDNA copy number and transcriptional coactivators peroxisome proliferator-activated receptor gamma coactivator 1-alpha and beta (Pgc1A and B) and the genes associated with them [8]. Further, the efficiency of energy metabolism is associated with the integrity of the proteins that constitute the electron transport chain (ETC) complexes. Shortcomings in the function or assembly of these complexes may result in deficits in energy production and mitochondrial functions. Among the five complexes that constitute the ETC, complex I is the largest multi-enzyme complex and is implicated in the pathogenesis of numerous hereditary and degenerative disorders [9,10]. Metabolic flexibility is a measure of the mitochondrial ability to switch between glucose and fatty acids as the fuel in response to the abundance of the specific substrate. The two major mitochondrial substrates are pyruvate and fatty acids. Efficient switching between them, depending on the substrate availability, is essential to maintain energy homeostasis during various metabolic states such as fasting, feeding, and exercise [6,11]. Metabolic inflexibility contributes to exercise intolerance and may be a factor in limiting participation in strenuous activities. For instance, skeletal muscle mitochondrial dysfunction and metabolic inflexibility were frequently seen in young individuals who have a sedentary lifestyle [12,13,14,15]. Mitochondrial pyruvate carrier protein1 (Mpc1) is the main transporter of pyruvate into the mitochondria. In addition, the pyruvate dehydrogenase complex enzymes (Pdha1) convert pyruvate into acetyl-CoA to be oxidized in the citric acid cycle [16,17,18]. However, fatty acid oxidation mainly depends on the fatty acid transport into mitochondria, and it is regulated by carnitine palmitoyl transferase (CPT). In the skeletal muscle, the isoforms Cpt1b and Cpt2, situated in outer and inner mitochondrial membranes, respectively, are the limiting factors of mitochondrial fatty acid metabolism [19].

Several animal and epidemiological studies indicated the importance of prenatal diet on the developmental origin of health and diseases. Especially, an adequate amount of prenatal protein is crucial in the metabolic health of the adult offspring [20,21,22,23,24]. Fetal organs’ growth and metabolism depend on maternal nutrition, and a maternal low-protein (LP) diet impacts several fetal organs at the expense of some organs such as the brain. Among the different organs affected, the skeletal muscle is most susceptible to maternal protein deficiency [25]. We developed a unique lean T2D rat model by LP diet in utero only. Offspring were glucose intolerant and insulin resistant at 4 months [26]. Interestingly, we found a sex-specific difference in the molecular mechanisms of insulin resistance in the skeletal muscle [27,28]. We also noticed mitochondrial dysfunction and reduced complex I protein in the skeletal muscle of the LP female offspring; moreover, LP skeletal muscle was filled with large abnormal mitochondria, and mitochondrial respiratory capacity was reduced compared to the control [29]. However, maternal LP effects on the mitochondrial dynamics and bioenergetics in the male skeletal muscle are not known. Therefore, in this present study, we investigated the effects of a prenatal LP diet on mitochondrial dynamics and its influences on mitochondrial bioenergetics in the skeletal muscle of male offspring.

## 2. Results

### 2.1. LP Programming Lowered the Expression of Mitochondrial Dynamics and Biogenesis Genes in the GS Muscle

**Figure 1:**
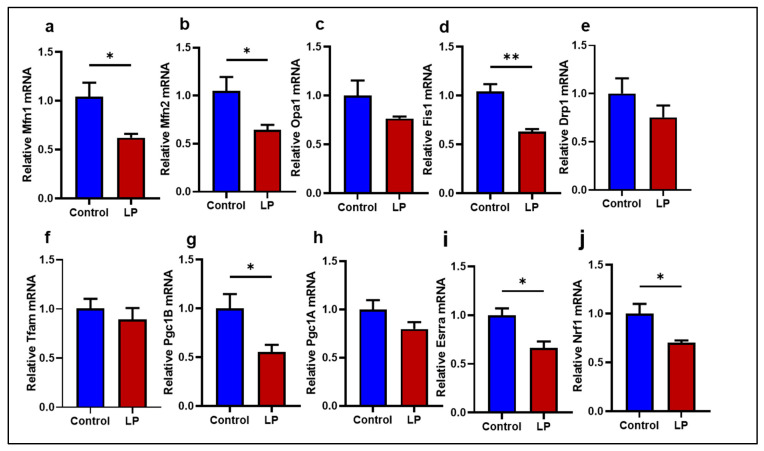
Effects of LP programming on the expression of genes involved in mitochondrial dynamics and biogenesis in the skeletal muscle of control and LP rats. The mRNA levels of mitochondrial dynamic genes: (**a**) Mfn1; (**b**) Mfn2; (**c**) Opa1; (**d**) Fis1; (**e**) Drp1 (**f**) Tfam, (**g**) Pgc1A (**h**) Pgc1B (**i**) Essra (**j**) Nrf1 were analyzed by qPCR. The mRNA expressions of each gene were normalized to the average of internal controls. Data represent mean ± SEM (* *p* < 0.05, ** *p* < 0.01); *n* = 5.

The expression of genes associated with mitochondrial dynamics and biogenesis was assessed to investigate the role of mitochondria in LP-programmed skeletal muscle. Overall, the skeletal muscle of LP offspring showed lower mRNA expression of mitochondrial fusion, fission, and biogenesis genes tested (Figure 1).

The outer mitochondrial membrane fusion genes, Mfn1 and Mfn2, levels were lower in the LP (*p* < 0.01 and *p* < 0.03) compared to the control (Figure 1a,b). However, the inner mitochondrial membrane fusion gene, Opa1, levels were not different from the control (Figure 1c). The mitochondrial fission gene, Fis-1, expression was severely reduced (*p* < 0.003) in the LP skeletal muscles compared to the control (Figure 1d), but no differences in the expression were observed in Drp1 (Figure 1e). Further, genes associated with mitochondrial biogenesis, such as Pgc1B (Figure 1g; *p* < 0.025), Esrra (Figure 1i; *p* < 0.02), and Nrf1 (Figure 1j; *p* < 0.03), were significantly lower in the LP group when compared to the control. However, the mitochondrial-biogenesis-associated gene Pgc1A expression in the LP was not significantly different from the control (Figure 1h). Similarly, Tfam, an activator of mitochondrial transcription and genome replication, was not significantly different between the groups (Figure 1f).

**Figure 2:**
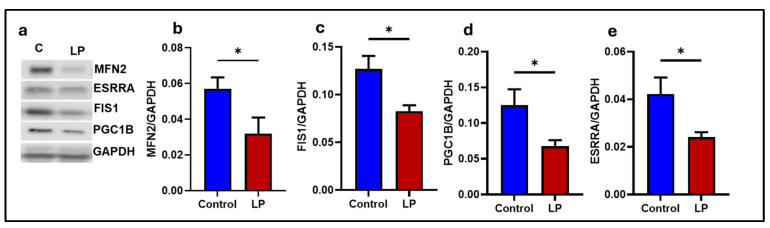
Effects of LP programming on levels of the proteins regulate mitochondrial fission, fusion, and biogenesis in the GS muscle of control and LP rats. (**a**) Representative WB. (**b**) MFN2; (**c**) FIS1; (**d**) PGC1B (**e**) ESSRA. The expressions of each protein were normalized to GAPDH expression. Data represent mean ± SEM (* *p* < 0.05); *n* = 5.

Further, the protein expression of the mitochondrial dynamics and biogenesis genes reflected the respective mRNA expression (Figure 2a). The protein levels of the mitochondrial outer membrane MFN 2 (*p* < 0.04) and fusion protein FIS-1 were significantly (Figure 2d, *p* < 0.01) reduced in the LP when compared to the control (Figure 2a–c). Further, the mitochondrial-biogenesis-linked gene PGC1B (*p* < 0.03) and ESRRA (*p* < 0.02) levels were significantly (*p* < 0.05) reduced in the LP compared to the control (Figure 2e).

### 2.2. The LP Programming Increased the mtDNA Copy Number in the GS Muscle

**Figure 3:**
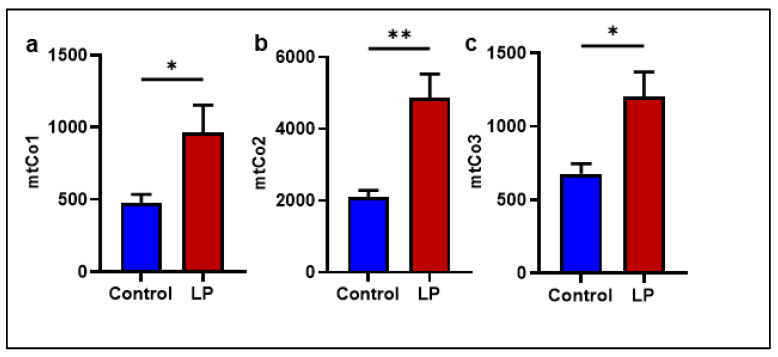
Effects of LP programming on mitochondrial DNA copy number in control and LP programmed groups as quantified using qPCR. (**a**) Mitochondrially encoded Cytochrome C Oxidase I (mtCo1) levels; (**b**) shows mtCo2 levels; (**c**) illustrate mtCo3 levels when normalized to beta-actin. Data represent mean ± SEM; (* *p* < 0.05, ** *p* < 0.01); *n* = 5.

The mtDNA copy number was quantified to assess the changes induced by LP programming in the mitochondrial genome. The mtDNA copy number was determined by the ratio between the expression of mitochondrial complex genes (mtCo1, mtCo2, and mtCo3) compared with a somatic reference gene (tubulin). Our data showed that the mtDNA copy numbers in the LP were significantly higher in all three complex genes (mtCo1, *p* < 0.031; mtCo2, *p* < 0.001 and mtCo3, *p* < 0.017) tested compared to the control (Figure 3a–c)

### 2.3. LP Diet-Induced Fetal Programming Leads to Reduced OCR in the Flexor Digitorum Brevis Muscle

**Figure 4:**
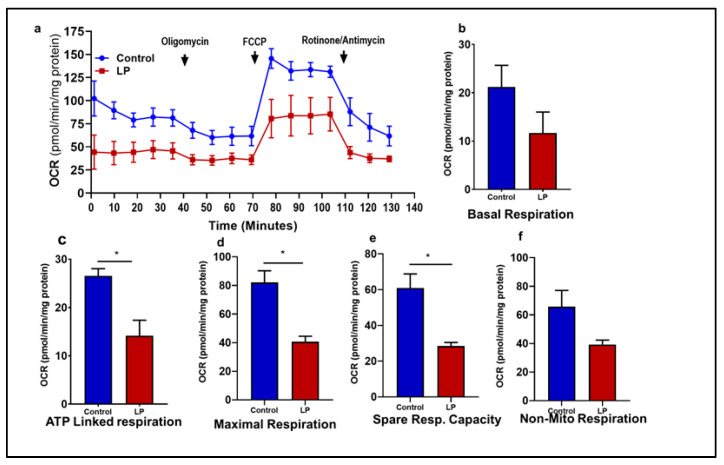
Effects of LP programming on mitochondrial oxygen consumption rate (OCR) in control vs. LP FDB muscle. The OCR measured before the addition of inhibitors was basal respiration. The arrows indicate the exact time at which different inhibitor compounds were injected into the wells: (**a**) Representative image of normalized mitochondrial OCRs; control (blue) vs. LP (red). (**b**) Basal respiration. (**c**) ATP-linked respiration. (**d**) Maximal respiration. (**e**) Spare respiratory capacity. (**f**) non-mitochondrial respiration. Data represent mean ± SEM (* *p* < 0.05); *n* = 3.

To examine the impact of maternal LP diet on offspring’s mitochondrial function, the change in the OCR was measured using the Agilent seahorse analyzer with a Mito Stress Test kit. Due to the large size of GS muscle fibers, culturing in the XF 96 Extracellular Flux Analyzer (Agilent, Santa Clara, CA 95051, USA) plate was not achieved; considering the mixed fiber nature of the GS muscle, we used the flexor digitorum brevis (FDB), a small muscle with a mixed fiber type, as an alternative. Figure 4a depicts the changes in the OCR at the basal level and after sequential addition of different inhibitors. Muscle fibers from the LP rats exhibited lower OCRs throughout the measurements compared to the controls with no significant changes (11.67 ± 2.8 pmol/min/mg protein in LP vs. 21.19 ± 2.8 controls) in the basal OCRs (Figure 4b). A similar trend was observed with the non-mitochondrial respiration (39.24 ± 2.0 pmol/min/mg protein in LP vs. 65.70 ± 7.2 in controls) (Figure 4f). In contrast, the LP programming caused a significant reduction (14.16 ± 2.0 pmol/min/mg protein in LP vs. 26.0 ± 0.9 in controls, *p* < 0.02) in ATP-linked OCRs (Figure 4c). Similarly, the maximal OCR (40.65 ± 2.4 pmol/min/mg protein in LP vs. 82.05 ± 5.2 in controls, *p* < 0.01) and spare respiratory capacity (28.42 ± 1.3 pmol/min/mg protein in LP vs. 60.89 ± 5.0 in controls; *p* < 0.01) exhibited lower OCRs in the LP when compared to the control group (Figure 4d,e).

### 2.4. Mitochondrial Complex I Genes Are Downregulated in LP GS Muscles

**Figure 5:**
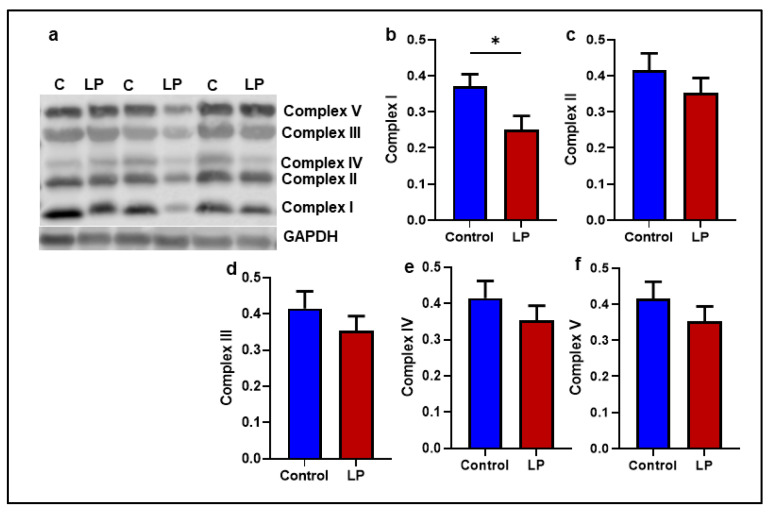
Effects of LP programming on mitochondrial ETC complexes protein levels in control vs. LP GS muscle: (**a**) Representative image of different mitochondrial complex protein content. (**b**–**f**) Fold-change of protein levels for the different subunits of mitochondrial complexes: (**b**) Complex I (CI, Ndufb8); (**c**) Complex II (CII, Sdhb); (**d**) Complex III (CIII, Uqccrc2); (**e**) Complex IV (CIV, mtCo); (**f**) Complex V (CV, ATP5a). Data represent mean ± SEM (* *p* < 0.05); *n* = 5.

**Figure 6:**
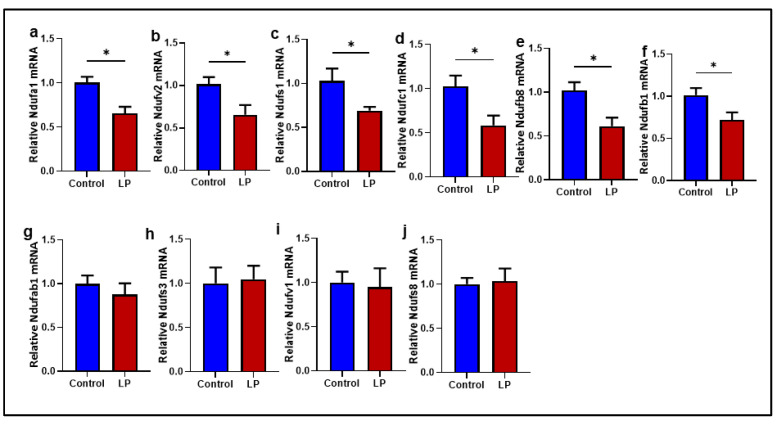
Effects of LP programming on the expression of key nuclear genes involved in the complex-1 function in the GS muscle of control and LP rats. The mRNA levels of mitochondrial dynamic genes: (**a**) Ndufa1; (**b**) Ndufv2; (**c**) Ndufs1; (**d**) Ndufc1; (**e**) Ndufb8 (**f**) Ndufb1, (**g**) Ndufab1 (**h**) Ndufs3 (**i**) Ndufv1 (**j**) Ndufs8 were analyzed by qPCR. The mRNA expressions of each gene were normalized to the average of internal controls. Data represent mean ± SEM (* *p* < 0.05), *n* = 5.

To analyze the effects of the LP programming on the electron transport chain (ETC), the levels of representative proteins associated with different complexes that constitute the electron transport chain were measured (Figure 5). Among the five different proteins tested, the expression of Ndufb8 (complex I) was 1.5-fold lower (*p* < 0.05) in the LP group compared to the control (Figure 5b). Although not statistically different, the levels of Sdhb (complex II), Uqcrc2 (complex III), mtCo1 (complex IV), and Atp5a1 (complex V) also showed a decreasing trend in the GS muscle of the LP group but not significantly different (Figure 5c–f). As a significant difference in the complex I protein was observed, further analysis was conducted to see the mRNA levels of key genes associated with complex I activity (Figure 6). Among the ten genes tested, NDUFA1 (*p* < 0.013), NDUFV2 (*p* < 0. 02), NDUFS1 (*p* < 0.04), NDUFC1 (*p* < 0.03), NDUFB8 (*p* < 0.02), and NDUFB1 (*p* < 0.03) were significantly lower in the LP compared to the control (Figure 6a–f). However, no significant differences were observed in the expression of the NDUFAB1, NDUFS3, NDUFV1, and NDUFS8 levels (Figure 6g–j).

### 2.5. The LP Diet Altered the Genes Associated with Mitochondrial Substrate Transport and Oxidation in the GS Muscle

**Figure 7:**
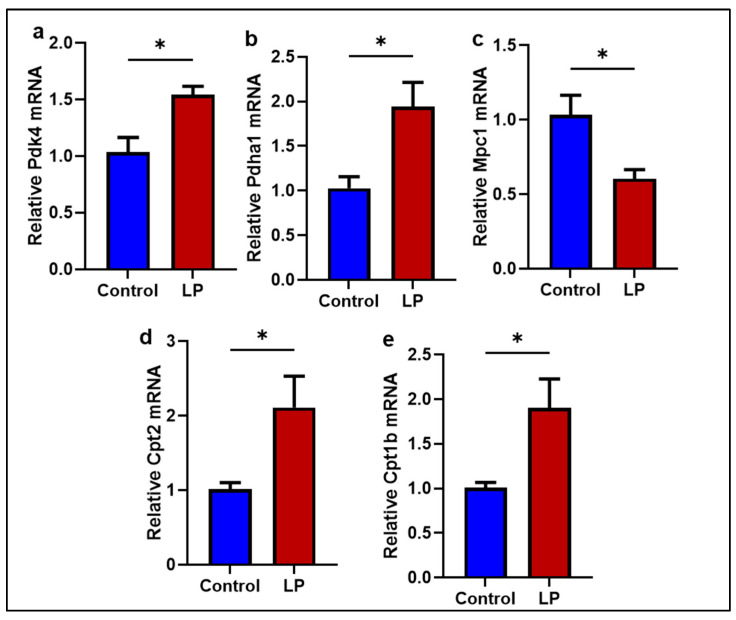
Effects of LP programming on gene expressions in pyruvate and fatty acid transport and metabolism in the GS muscle of control and LP rats. The mRNA levels of mitochondrial dynamic genes: (**a**) Pdk4; (**b**) Pdha1; (**c**) Mpc1; (**d**) Cpt2; and (**e**) Cpt1b were analyzed by qPCR. The mRNA expressions of each gene were normalized to average internal controls. Data represent mean ± SEM (* *p* < 0.05); *n* = 5.

**Figure 8:**
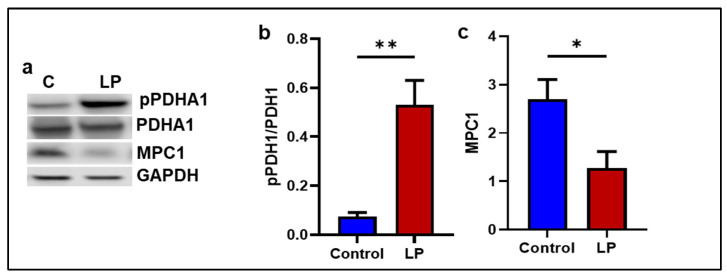
Effects of LP programming on the expression proteins involved in mitochondrial pyruvate transport and metabolism in the GS muscle of control and LP rats. (**a**) Representative WB images. (**b**) pPDHA1/PDHA1; (**c**) MPC1; The expressions of each protein were normalized to GAPDH expression. Data represent mean ± SEM (* *p* < 0.05, ** *p* < 0.01); *n* = 5.

To assess the effects of the LP diet on mitochondria, mRNA levels of genes involved in fatty acid and pyruvate transport and oxidation were measured. The pyruvate oxidation inhibitor Pdk4 was higher in the LP muscle, and the pyruvated transporter Mpc1 levels in the LP muscle were lower (*p* < 0.05) compared to the control group (Figure 7a,c). However, Pdha1, the expression of a key gene that facilitates pyruvate metabolism, was not different between the groups (Figure 7b). Further, the mitochondrial fatty acid transporter Cpt2 and Cpt1b mRNA levels were significantly higher (*p* < 0.05 in the LP group) compared to the control (Figure 7d,e). Next, the protein levels of the genes associated with pyruvate and fatty acid transport to mitochondria were determined (Figure 8a). The ratio of pPDHA1 to total PDHA1 in the LP was higher compared to the control (Figure 8b). Conversely, the MPC1 protein levels were lower in the LP group (Figure 8c).

### 2.6. LP Diet Did Not Alter the Mitochondrial Morphology in the GS Muscle of the Offspring

**Figure 9:**
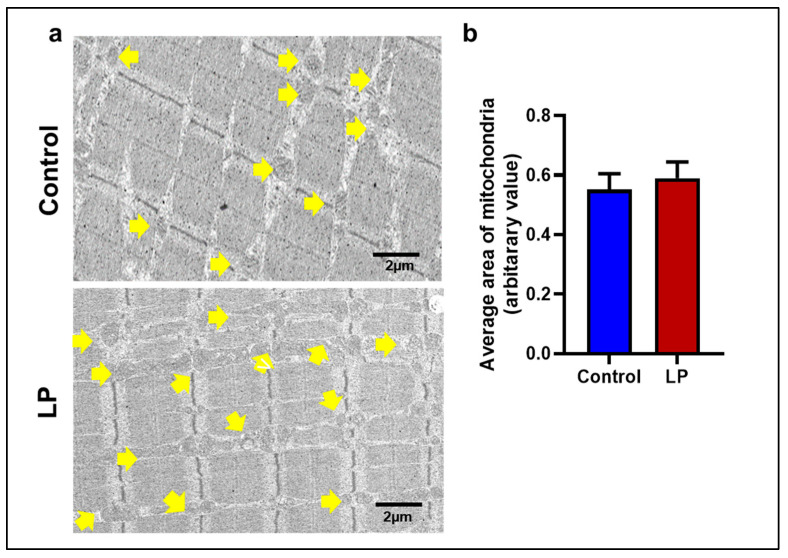
The LP diet altered the mitochondrial morphology in the GS muscle of the offspring. (**a**) Representative TEM images showing changes in mitochondrial morphology (arrow) (**b**) A graph showing the average area (10 images/animal) of mitochondria in the GS muscle. *n* = 5.

Considering the role of healthy mitochondria in glucose metabolism in the GS muscle, the morphology and ultrastructure of the mitochondria in the GS muscle of LP-programmed lean T2D rats were assessed. Although more abnormally large mitochondria were observed in the LP group, image analysis did not show statistical significance between the groups. No significant differences were noticed by analyzing the total and mean area covered by the mitochondria between the groups (Figure 9).

## 3. Discussion

Mitochondria play a crucial part in insulin-dependent glucose disposal and glucose homeostasis in the skeletal muscles, and the efficiency of glucose disposal is largely dependent on insulin sensitivity and the quality of mitochondria. The in-utero LP exposure led to fetal growth restriction and lower body weight in pups; however, they caught up with growth by 2 months of age (Supplementary Figure S1). In our previous studies, we demonstrated that LP-programmed T2D rats had impaired insulin signaling, resulting in glucose intolerance and mitochondrial dysfunction in the GS muscles of female offspring [27,30].

Mitochondria are dynamic organelles and assume different shapes and sizes depending on the cell type and microenvironment. They maintain their dynamic nature by a combination of fission, fusion, and biogenesis [31,32]. Since the dynamic nature of mitochondria is essential for its normal functions, even minor irregularities in the dynamics might lead to mitochondrial dysfunction [33]. Moreover, impaired mitochondrial dynamics can disrupt normal glucose metabolism and cause insulin resistance [34]. Unusual mitochondrial dynamics are commonly associated with irregular mitochondrial fission, fusion, or biogenesis [4]. Our data show that the expression of genes associated with mitochondrial outer membrane fusion, such as Mfn1 and Mfn2 levels, were lower in the LP group compared to controls, indicating that the mitochondrial fusion process is impacted by the LP programming in the male rats. We have earlier reported similar results in the skeletal muscle of LP-programmed females [29]. Previous studies have shown a clear link between the downregulation of mitochondrial fusion genes in the skeletal muscles and dysregulated glucose metabolism in type 2 diabetic animal models and humans [35,36,37,38,39].

In the present study, we report that the LP programming results in lower expression of genes linked to mitochondrial dynamics and biogenesis (Figure 1 and Figure 2) along with dysregulated ETC complex I genes (Figure 5 and Figure 6) in the LP-programmed male rats. Interestingly, the mtDNA copy numbers were higher in the LP rats compared to the controls. In the fed state, LP programming altered mitochondrial substrate preference, increasing reliance on fatty acids due to higher expression of mitochondrial fatty acid transporters (Figure 7d, e). Conversely, it inhibited genes related to pyruvate transport and metabolism (Figure 7a–c and Figure 8). As a result, mitochondrial function in LP skeletal muscle may be compromised during periods of low fatty acid availability. This reduction in mitochondrial function is reflected in the decreased overall oxygen consumption observed in the FDB muscle (Figure 3). However, the total skeletal muscle weights were not different between the control and LP groups (Supplementary Figure S2).

The mitochondrial fission gene Fis1 was downregulated in the LP group compared to the controls, but Drp1, another key player in the fission, was unchanged. Fis1 is a mitochondrial surface protein that helps to recruit Drp1 onto the mitochondrial surface to initiate fission [40]. Further, no change was observed in the expression of Opa1, which is responsible for the inner mitochondrial membrane fusion [41]. Taken together, our data indicates that the LP diet programming only affected the molecules present on the outer rather than the inner membrane of the mitochondria. Consequently, the abnormal mitochondrial morphology is not significantly different in the TEM images of the LP group (Figure 9 and Supplementary Figure S3). In addition, the downregulation of the PGC-1 family regulatory network involved in mitochondrial biogenesis, such as Pgc1 B, Nrf1, and Esrra levels, indicate that the mitochondrial biogenesis was lower in LP males in comparison to the controls indicating an altered homeostasis of mitochondrial dynamics [42]. Although the nuclear genes associated with mitochondrial proteins are affected due to LP programming, the expression of Tfam, which regulates the mtDNA replication, was not different, indicating that the mtDNA replication is unaffected. However, the mtDNA copy number in the skeletal muscle of LP offspring was higher, suggesting dysregulated mitochondrial quality control [43,44,45]. The increase in the mtDNA copy number is shown to be a part of a compensatory mechanism due to the presence of mutated mtDNA in the dysfunctional mitochondria [46]. Thus, the higher mtDNA copy number seen in the LP muscle could be to compensate for the presence of dysfunctional mitochondria rather than the cause of dysfunction.

Abnormal mitochondrial dynamics and reduced biogenesis in the GS suggest the presence of dysfunctional mitochondria and could cause reduced energy production [47,48,49,50]. In addition, dysregulated mitochondrial dynamics in skeletal muscle, particularly impairments in fission protein Fis1 and fusion protein Mfn2, lead to structural and functional mitochondrial abnormalities. When mitochondrial dynamics are compromised, skeletal muscle mitochondria may exhibit disrupted biogenesis, reduced respiratory function, and decreased bioenergetic efficiency, impairing the muscle’s ability to meet high energy demands. Hence, we investigated the mitochondrial functions to identify changes to mitochondrial OCRs due to the LP programming using a Mito Stress Test. The LP males exhibited an overall lower OCR at the different stages of measurements, indicating the presence of dysfunctional mitochondria in the skeletal muscle. To be precise, the OCR linked to ATP production was significantly lower in the LP group compared to the controls. As ATP production is the main function of the mitochondria ETC complex, the lower ATP-linked OCR indicates an inefficient ETC complex in these rats. In addition, the lower basal OCR indicates the incompetence of the LP-programmed mitochondria to meet the basic ATP demand in the muscle. Lower OCRs in the maximal respiratory capacity and spare respiratory indicate that the mitochondrial inner membrane integrity is compromised in the LP group. Although mitochondria are the major oxygen consumer, several cellular processes, such as DNA and histone demethylation and lipid and collagen synthesis, consume oxygen, which is known as non-mitochondrial oxygen consumption [51]. The lower levels of non-mitochondrial OCRs indicate that the LP programming impaired normal cellular processes in the skeletal muscle. Our previous study in the LP-programmed female offspring also exhibited similar dysfunctional mitochondria [29]. Moreover, previous clinical studies reported a lower mitochondrial respiration rate in the skeletal muscle of T2D patients [52,53,54].

Interestingly, the mtDNA copy number was higher in the skeletal muscle of the LP group compared to the controls. A higher mtDNA copy number is often associated with improved mitochondrial function [55]. However, in our study, the increased mtDNA copy number did not improve the mitochondrial quality, which indicates the LP programming-induced impact on mitochondrial health was not restored in the skeletal muscles. The increase in the mtDNA copy numbers could be an attempt in the LP-programmed group to compensate for the inefficiency of the mitochondria. Our previous studies in female rats showed no significant differences in the mtDNA copy number; however, the mitochondrial structure was altered with the loss of cristae and an increase in mitochondrial length [29]. In the present study in males, we did not find any significant changes in the mitochondrial structure and length in the GS muscle of LP-programmed males when compared to the controls, strongly suggesting sex differences in LP programming [29,56]. Further, these data suggest that the nuclear proteins associated with mitochondrial energetics might be the initiator of LP-programmed mitochondrial dysfunction in the male rats, rather than mtDNA-coded proteins [57,58,59].

The integrity of the electron transport chain (ETC) complex proteins present at the inner mitochondrial membrane is a direct gauge of mitochondrial function and quality [60,61,62]. Hence, we determined the levels of key proteins in the different ETC complexes. Our data showed that LP diet programming caused a significant reduction in the levels of Nduf8, one of the critical catalytic proteins present in the complex I of the ETC. We have reported lower Nduf8 levels previously in the LP-diet-programmed female offspring skeletal muscle and liver [63]. We further investigated the mRNA expression of key genes associated with the catalytic activity of complex I and observed that most genes tested exhibited lower levels in the LP group, indicating LP programming somehow affected the expression of complex I genes. Similarly, other groups have also observed a strong correlation between lower ETC gene expression and mitochondrial dysfunction [62,64]. Complex I is essential for NADH oxidation and mitochondrial super complex formation. Several mitochondrial disorders, such as exercise intolerance, Leigh syndrome, and lactic acidosis, are the result of complex I deficiency [57]. Further, the skeletal muscles of type 2 diabetic patients lack mitochondrial super complex-I formation, leading to mitochondrial dysfunction [57,65]. Thus, the lower levels of mRNA and proteins associated with complex I catalytic subunits observed in the LP males could indicate the loss of integrity of complex I, thereby causing mitochondrial dysfunction.

The LP-induced loss of complex-I integrity and mitochondrial dysfunction was further evident in the alteration of genes associated with substrate oxidation in the GS muscle. The LP group showed higher expression of fatty acid transporter, Cpt1b, and Cpt2, and lower expression of mitochondrial pyruvate transporter, Mpc1, indicating fatty acid preference over pyruvate [66,67,68]. The higher levels of Pdk4, an inhibitor of pyruvate metabolizing enzyme Pdah1, and pPdha1, further suggest that LP-exposed mitochondria might prefer fatty acids over pyruvate as a substrate for energy production [69,70,71]. The mitochondrial inability to use glucose and fatty acids efficiently may lead to elevated levels of glucose in circulation and could be the reason for glucose intolerance seen in these animals [26,72]. Eventually, this metabolic gridlock may lead to metabolic inflexibility and impaired mitochondrial dynamics [6,11,73]. Therefore, the loss of complex-I integrity and associated impaired mitochondrial metabolism might be one of the main causes of impaired mitochondrial dynamics and function in the skeletal muscle of LP males.

In summary, our results indicate that low-protein programming affects the expression of genes associated with mitochondrial dynamics and biogenesis. Further, the integrity of mitochondrial complex-I was compromised in the gastrocnemius muscle. These low-protein programming-mediated effects resulted in alteration in genes associated with mitochondrial substrate oxidation and lower bioenergetics, leading to mitochondrial dysfunction in the skeletal muscles. This mitochondrial dysfunction might be a major driver of lean T2D observed in these rats. Therefore, efforts to improve mitochondrial health in the skeletal muscle might be a viable strategy to prevent low-protein-induced leanT2D.

## 4. Materials and Methods

### 4.1. Animals

All the methods are reported following ARRIVE guidelines for the reporting of animal experiments. All the studies agreed with the National Institutes of Health guidelines for the Care and Use of Laboratory Animals. The procedures were approved by the Institutional Animal Care and Use Committee of Baylor College of Medicine, Houston, Texas. Outbred Wistar rats were obtained from Envigo Bioproducts Inc. Madison, WI, USA. Virgin females weighing ~250 g and males weighing ~350 g were purchased and housed in a temperature-controlled room (~23 °C) with a 10:14 h light/dark cycle and were given unlimited access to food and water. Female rats were mated with males of proven fertility by housing two females with one male. The females were checked for the presence of sperm in the vaginal smear, and the presence of sperm was marked as day one of pregnancy. On day four of the pregnancy, rats were randomly assigned to a control diet containing 20% protein (*n* = 10) or an isocaloric 6% protein-containing diet (*n* = 10) (Harlan Teklad, Madison, WI, USA) until delivery. Standard laboratory rat chow (Teklad Global 2019, Teklad Diets, Madison, WI, USA) was given to dams after delivery until the end of weaning, and pups were given the standard laboratory rat chow after weaning. Two-day-old pups were sexed, and pups with extreme weights were culled to normalize the litter size to 8 pups (4 males and 4 females when possible) per mother in both groups. All experiments were performed using 4-month-old rats except for the Seahorse XF cell Mito Stress Test, where the rats were 5 months old. Rats were euthanized by CO_2_ asphyxiation followed by bilateral thoracotomy at the diestrus stage, and tissues were harvested and processed for various studies. All the tissues were collected at fed condition.

### 4.2. Transmission Electron Microscopy (TEM)

TEM was carried out as described previously [74]. Briefly, gastrocnemius muscles were dissected and placed in fixative (2.5% glutaraldehyde in 0.1 M cacodylate buffer to a pH of 7.4 at 4 °C) and trimmed into small pieces. The pieces were placed in the fixative and stored at 4 °C until processing. The muscle pieces were then washed for 45 min with gentle shaking in cacodylate buffer with changes every 15 min, followed by post-fixing in 1% OsO_4_ and in 0.1 M cacodylate for 45 min at 4 °C. The samples were washed three times in distilled water and washed in an ascending series of alcohol. The muscle samples were then embedded and cured in resin. Then, 50 nm sections were cut from these blocks using an ultra-microtome. The thin sections were mounted on a copper grid and stained with heavy metals for ultra-structural analysis.

### 4.3. Mitochondrial DNA Copy Number

Total DNA was isolated from gastrocnemius muscle using a QIAmp DNA kit (Qiagen, Hilden,Germany). The genomic DNA was stored at −80 °C for subsequent use. The mitochondrial DNA copy number was assessed as previously described [74,75]. The quantitative real-time (qRT) was performed with 1:100 diluted DNA templates for mitochondrially encoded cytochrome c oxidase 1, 2, and 3 and the nuclear genes tubulin and beta actin. The total reaction volume was 10 μL, and the reaction mix was prepared fresh every time. The PCR conditions were 3 min at 95 °C for initial denaturation, followed by 15 s at 95 °C, 30 s at 60 °C for annealing, and 15 s at 72 °C for the extension for 40 cycles, followed by a melt curve analysis. All samples were run in triplicates. Details of the primers are provided in Table 1.

### 4.4. Quantitative Real-Time (qRT)-PCR

The expression of key genes involved in mitochondrial dynamics and function was quantified using qPCR. Total RNA was isolated from gastrocnemius muscle by using TRIzol reagent (Life Technologies, Carlsbad, CA, USA). Total RNA was further refined with the RNeasy clean-up kit (Qiagen, Valencia, CA, USA). All the RNA samples were quantified using an ND-1000 model Nanodrop spectrophotometer (Thermo Fisher Scientific, Newark, DE, USA). Total RNA (2 µg) was reverse transcribed using a modified Maloney murine leukemia virus-derived RT (New England Biolabs Inc., Ipswich, MA, USA) and random hexamer primers (Life Technologies, Carlsbad, CA, USA) as reported earlier [27]. After dilution, cDNA was amplified by real-time PCR using SYBR Green (Bio-Rad, Hercules, CA, USA) in a CFX96 model real-time thermal cycler (Bio-Rad). Specific pairs of primers were designed and purchased (IDT, Coralville, IA, USA). Details of primers are provided in Table 1. All reactions were performed in triplicates, and the averages of cyclophilin A, beta-actin, and GAPDH were used as internal references. The results were calculated using the 2^−ΔΔCT^ method and expressed as fold changes in gene expression for the genes of interest.

### 4.5. Mitochondrial Oxygen Consumption

FDB muscles were isolated, and the Mito Stress Test was performed using an XF 96 Extracellular Flux Analyzer (Seahorse Bioscience, Agilent, Santa Clara, CA 95051, USA) as described earlier [76]. Briefly, the FDB muscle (~20 μg) from each rat was incubated in dissociating media at 5% CO_2_ and 37 °C for 2 h. After dissociation, single myofibers were separated from each FDB muscle bundle. After removing the undigested fragments, myofibers were transferred to a 35 mm sterile dish with 1 mL of culture media. After thoroughly dispersing the muscle fibers, 50 μL aliquots of the fibers were taken and seeded into the Aligent Seahorse XFe96 microplate pre-coated with extracellular matrix (ECM, Sigma, St. Louis, MO 68178, USA) to facilitate attachment of the muscle fibers. Samples from each animal were seeded in triplicates. The microplates were placed in a 5% CO_2_ incubator at 37 °C overnight before analysis. Before the assay, the culture medium was replaced with Seahorse XF Assay Medium (Agilent, Santa Clara, CA 95051, USA) supplemented with 10 mM glucose, 1 mM pyruvate, and 2 mM glutamine, pH 7.4. Before initiating the assay, the sensor cartridge was loaded with injection compounds: Oligomycin, Carbonyl cyanide-4-(trifluoromethoxy) phenylhydrazone (FCCP), and rotenone + antimycin A). Mitochondrial stress tests followed the manufacturer’s protocol (Agilent Technologies).

### 4.6. Western Blot

Western blots for mitochondrial proteins in gastrocnemius muscle were performed as described earlier [77]. Briefly, 30 μg of protein extract was resolved on 4–15% precast gradient polyacrylamide gels (Mini-PROTEANTGX Precast Gels; Bio-Rad, Hercules, CA, USA). Resolved proteins were transferred to a polyvinylidine fluoride membrane (Millipore, Billerica, MA, USA). Primary antibodies were incubated overnight at 4 °C after blocking the membranes in 5% bovine serum albumin or 1% nonfat dried milk in Tris-buffered saline containing 0.1% Tween 20 for 1 h at room temperature. Details of primary antibodies and their dilutions are as follows: and Gapdh (Cat #97166, 1:1000), Vdac1 (Cat #4661, 1:1000), Opa1 (Cat #80471, 1:1000), Nrf1 (Cat #46743, 1:1000), Sirt1 (Cat #9475, 1:1000), Erra (Cat #13826, 1:1000), Cox-IV (Cat #4850, 1:5000) were obtained from cell signaling, Danvers, MA, USA; Fis1 (Cat # sc-376447, 1:1000), Mfn2 (Cat # sc-515647, 1:1000) were obtained from Santa Cruz Biotechnology, Dallas, TX, USA. Total OXPHOS rodent antibody cocktail (Cat # ab110413), Pgc1b (Cat # ab 176328) were obtained from Abcam Cambridge, MA, USA, respectively. After primary antibody incubations, membranes were washed and incubated for 1 h at room temperature with horseradish peroxidase-conjugated secondary antibodies (Proteintech Inc. Rosemont, IL, USA). Membranes were washed and incubated in ECL Western blotting detection reagents (Pierce Biotechnology, Waltham, MA, USA) for detection and imaged using the Odyssey Fc imaging system (LI-COR Biotechnology, Lincoln, NE, USA). Densitometry analyses were performed using Image Studio version 6.0 (https://www.licor.com/bio/image-studio) (accessed on 10 January 2024) (LI-COR Biotechnology, Lincoln, NE, USA).

### 4.7. Statistical Analysis

Statistical analyses were performed using GraphPad Prism 10.1 software ((accessed on 22 March 2024) (GraphPad, La Jolla, CA, USA). Data are presented as the mean ± SEM. Comparisons between the two groups were performed using unpaired Student *t*-tests. Differences were statistically significant at *p* < 0.05.

## Figures and Tables

**Table 1:** Oligonucleotide primers used for real-time PCR.

Gene	Accession NO:	Primers F = Forward; R = Reverse	Amplicon Size (bp)
mtCox1	MW209726.1	F: 5′-ATCGCAATTCCTACAGGCGT-3′ R: 5′-TGTTAGGCCCCCTACTGTGA-3′	129
mtCox2	MW209726.1	F: 5′-CAAGACGCCACATCACCTATC-3′ R: 5′-TTGGGCGTCTATTGTGCTTG-3′	150
mtCox3	MW209726.1	F: 5′-GGAACATACCAAGGCCACCA-3′ R: 5′-TCGTGGGTAGGAACTAGGCT-3′	140
Esrra	NM_001008511.2	F: 5′-AAAGTCCTGGCCCATTTCTATG-3′ R: 5′-CCCTTGCCTCAGTCCATCAT-3′	101
Drp1	NM_053655.3	F: 5′-CTGGTCCACGTTTCACCAGA-3′ R: 5′-CCCATTCTTCTGCTTCAACTCC-3′	73
Tfam	NM_031326.2	F: 5′-TCGCCTGTCAGCCTTATCTG-3′ R: 5′-TTACATCTGGGTGTTTAGCTT-3′	133
Cyclophilin A	XM_006250801.5	F: 5′-TATCTGCACTGCCAAGACTGAGTG-3′ R: 5′-CTTCTTGCTGGTCTTGCCATTCC-3′	127
Fis1	NM_001105919.	F: 5′-GTGCCTGGTTCGAAGCAAATA-3′ R: 5′-CATATTCCCGCTGCTCCTCTT-3′	101
Mfn1	NM_138976.2	F: 5′-ATCTTCGGCCAGTTACTGGAGTT-3′ R: 5′-AGATCATCCTCGGTTGCTATCC-3′	101
Mfn2	NM_001429969.1	F: 5′-CCTTGAAGACACCCACAGGAATA-3′ R: 5′-CGCTGATTCCCCTGACCTT-3′	101
Nrf1	NM_001100708.1	F: 5′-CTCTGCATCTCACCCTCCAAAC-3′ R: 5′-TCTTCCAGGATCATGCTCTTGTAC-3′	101
OPA1	NM_133585.3	F: 5′-AAAAGCCCTTCCCAGTTCAGA-3′ R: 5′-TACCCGCAGTGAAGAAATCCTT-3′	101
Pgc1a	NM_031347.1	F: 5′-CTACAATGAATGCAGCGGTCTT-3′ R: 5′-TGCTCCATGAATTCTCGGTCTT-3′	101
Pgc1b	NM_176075.3	F: 5′-TCGGTGAAGGTCGTGTGGTATAC-3′ R: 5′-GCACTCGACTATCTCACCAAACA-3′	101
Beta actin	V01217.1	F: 5′-CCACCATGTACCCAGGCATT-3′ R: 5′-GCTGACCACACCCCACTATG-3′	119
Tuba1a	XM_063263380.1	F: 5′-ATGGTCTTGTCGCTTGGCAT-3′ R: 5′-CCCCTTTCCACAGCGTGAGT-3′	135
Ndufa1	NM_001108813.2	F: 5′-GGGGGCAAGGAAAAGAGAGT-3′ R: 5′-CAGAGATGCGTCTATCGCGT-3′	73
Ndufv2	NM_031064.2	F: 5′-AGCCAGTTGGGAAGTACCAC-3′ R: 5′-CCCAGCTTTCTCTGAAGGGT-3′	97
Ndufs1	NM_001005550.1	F: 5′-CCAAGTGTGTCAAAGCCGTC-3′ R: 5′-TGTCCGTAGCAAAACAGGGT-3′	96
Ndufc1	NM_001399603.1	F: 5′-GTACTGCGCTCGTTTTCGC-3′ R: 5′-GTTTGGCATTGACTGGCTCC-3′	100
Ndufb8	NM_001106360.3	F: 5′-AGGCGGTGATCCTTCCAAAG-3′ R: 5′-GAGTCCCATTCAGAGGGCAC-3′	91
NdufAb1	NM_001106294.1	F: 5′-GGCTGCTGACTGGAACTTACT-3′ R: 5′-TTTGGGGCCAAATCTTCAGC-3′	100
Ndufb1	NM_001402546.1	F: 5′-CCTATGGGATTCGCCTTTGGA-3′ R: 5′-TTATTCCGGAAGGCAGTGAGC-3′	71
Ndufs3	NM_001106489.1	F: 5′-ATTTCCACTTCCGGTCCGTG-3′ R: 5′-CATGTTCCTTAGGGTGCCGA-3′	83
Ndufv1	NM_001006972.1	F: 5′-ACCTCATTTGGCTCGCTGAA-3′ R: 5′-CCTTCAGCCTCCAGTCATGG-3′	76
Ndufs8	NM_001106322.2	F: 5′-GAGCCGCTGCACTTCAAGAT-3′ R: 5′-GGCCATTAAGATGTCCTGTGC-3′	91
Pdk4	NM_053551.2	F: 5′-AGCAGTAGTCGAAGATGCCT-3′ R: 5′-CACGATGTGGATTGGTTGGC-3′	124
Pdha1	NM_001004072.2	F: 5′-GCAGCCAGCACGGATTACTA-3′ R: 5′-TCAGGATAGGCCCCTTACCA-3′	136
Mpc1	NM_133561.2	F: 5′-CGCAAAGCAGCGGACTATGT-3′ R: 5′-GGGCCCCAGAAGTGCGTA-3′	71
Cpt2	NM_001429335.1	F: 5′-CTAAGAGATGCTCCGAGGCG-3′ R: 5′-GGTCAGCTGGCCATGGTATT-3′	104
Cpt1b	NM_013200.2	F: 5′-CGAGTTCAGAAACGAACGCC-3′ R: 5′-TGGTGTGTCTCCTGGTCTCA-3′	115

## Data Availability

The data presented in this study are available on request from the corresponding author.

## Supplementary Materials

The following Supporting Information can be downloaded at: https://www.mdpi.com/article/10.3390/ijms252312860/s1.
