# Supplementary material for: Maternal Low-Protein Diet Leads to Mitochondrial Dysfunction and Impaired Energy Metabolism in the Skeletal Muscle of Male Rats
Source: Int J Mol Sci. 2024 Nov 29;25(23):12860. doi: 10.3390/ijms252312860 (PMC11641076; doi:10.3390/ijms252312860)
Supplement: Supplementary file 1 [file ijms-25-12860-s001.zip › ijms-3294145-supplementary.pdf]

Supplementary Figures for

Maternal Low Protein Diet Leads to Mitochondrial Dysfunction and Impaired Energy Metabolism in the Skeletal Muscle of Male Rats.

Vipin A. Vidyadharan1, Ancizar Betancourt1, Craig Smith2, Chellakkan S. Blesson3,4, and Chandra Yallampalli1*.

Copyright: © 2024 by the authors. Submitted for possible open access publication under the terms and conditions of the Creative Commons Attribution (CC BY) license (https://creativecommons.org/licenses/by/4.0/).

1 Basic Sciences Perinatology Research Laboratories, Department of Obstetrics and Gynecology, Baylor College of Medicine, Houston, Texas 77030, USA.

2 Agilent Technologies Inc., USA.

3 Reproductive Endocrinology and Infertility Division, Baylor College of Medicine.

4 Family Fertility Center, Texas Children's Hospital, Houston 77030, Texas, USA.

* Correspondence:

Chandra Yallampalli, Ph.D.

Professor and Director

Basic Sciences Perinatology Research Laboratories

Department of Obstetrics and Gynecology

Baylor College of Medicine

1102 Bates Street, Suite # 1850

Houston, Texas 77030

Office: 832-824 4188

Fax: 832-825 7946

Email: cyallamp@bcm.edu

Supplementary Figure S1

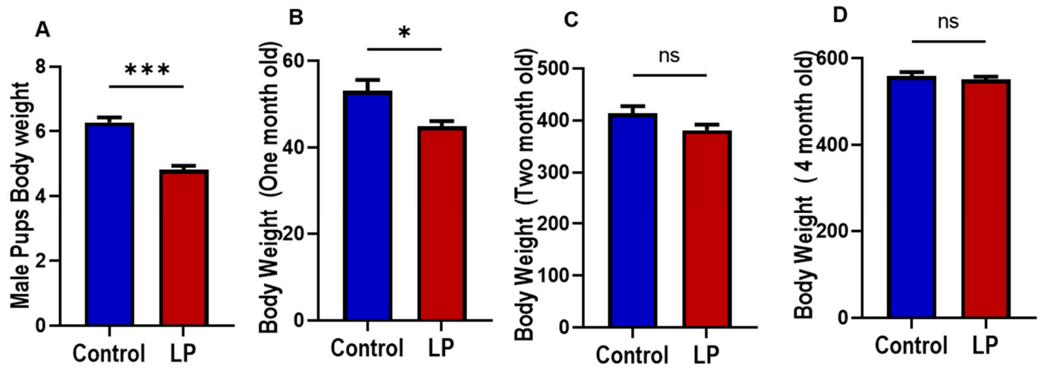

Fig.S1. Body weight of control and LP male offspring from birth to 4 months old. Data represent mean ± SEM (* $p<0.05$, *** $p<0.001$);) n=6-20.

Supplementary Figure S2

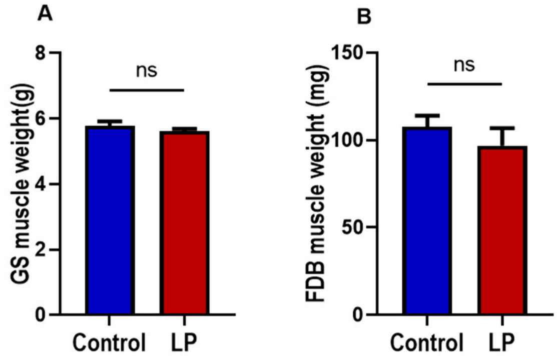

Fig.S2. Weight of skeletal muscle control and LP male offspring at 4 months old. A, GS muscle and B, FDB muscle. Data represent mean ± SEM , n=5.

Supplementary Figure S3

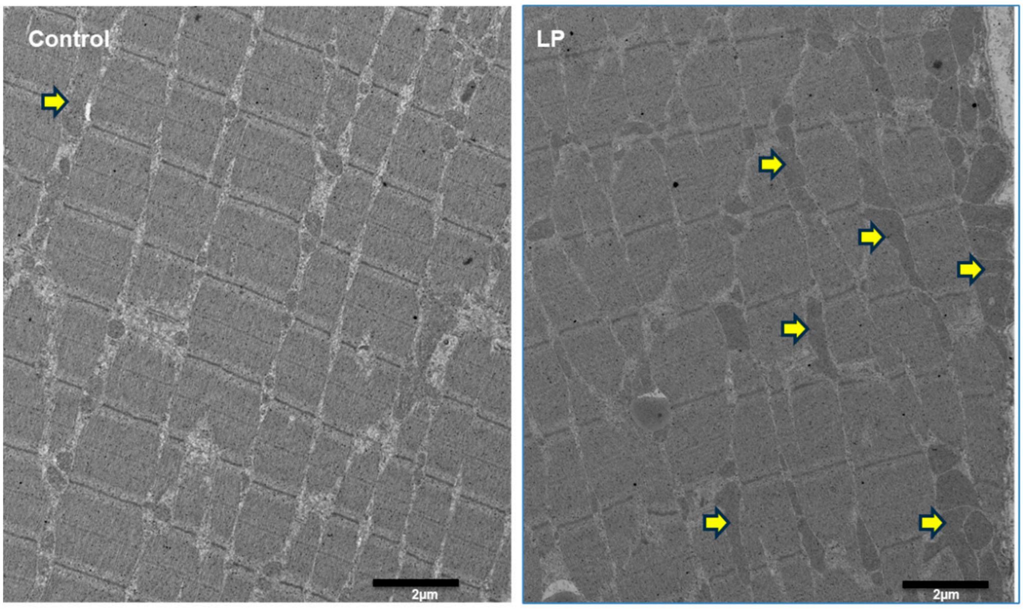

Fig.S3. The LP diet altered the mitochondrial morphology in the GS muscle of the offspring. Representative TEM images showing abnormal mitochondrial morphology (arrow). n=5.
